# A qualitative exploration of young people’s mental health needs in rural and regional Australia: engagement, empowerment and integration

**DOI:** 10.1186/s12888-023-05209-6

**Published:** 2023-10-13

**Authors:** Christiane Klinner, Nick Glozier, Margaret Yeung, Katrina Conn, Alyssa Milton

**Affiliations:** 1 Central Clinical School, Faculty of Medicine and Health, The University of Sydney, Sydney, Australia; 2 Australian Research Council (ARC), Centre of Excellence for Children and Families over the Life Course, Sydney, Australia; 3 NSW Department of Education, NSW, Australia

**Keywords:** Youth mental health, High school students, Help-seeking, Mental health education and support, Rural and regional Australia, Qualitative research

## Abstract

**Background:**

Australian rural and regional communities are marked by geographic isolation and increasingly frequent and severe natural disasters such as drought, bushfires and floods. These circumstances strain the mental health of their inhabitants and jeopardise the healthy mental and emotional development of their adolescent populations. Professional mental health care in these communities is often inconsistent and un-coordinated. While substantial research has examined the barriers of young people’s mental health and help-seeking behaviours in these communities, there is a lack of research exploring what adolescents in rural and regional areas view as facilitators to their mental health and to seeking help when it is needed. This study aims to establish an in-depth understanding of those young people’s experiences and needs regarding mental health, what facilitates their help-seeking, and what kind of mental health education and support they want and find useful.

**Method:**

We conducted a qualitative study in 11 drought-affected rural and regional communities of New South Wales, Australia. Seventeen semi-structured (14 group; 3 individual) interviews were held with 42 year 9 and 10 high school students, 14 high school staff, and 2 parents, exploring participants’ experiences of how geographical isolation and natural disasters impacted their mental health. We further examined participants’ understandings and needs regarding locally available mental health support resources and their views and experiences regarding mental illness, stigma and help-seeking.

**Results:**

Thematic analysis highlighted that, through the lens of participants, young people’s mental health and help-seeking needs would best be enabled by a well-coordinated multi-pronged community approach consisting of mental health education and support services that are locally available, free of charge, engaging, and empowering. Participants also highlighted the need to integrate young people’s existing mental health supporters such as teachers, parents and school counselling services into such a community approach, recognising their strengths, limitations and own education and support needs.

**Conclusions:**

We propose a three-dimensional *Engagement, Empowerment, Integration* model to strengthen young people’s mental health development which comprises: 1) maximising young people’s emotional investment (engagement); 2) developing young people’s mental health self-management skills (empowerment); and, 3) integrating mental health education and support programs into existing community and school structures and resources (integration).

**Supplementary Information:**

The online version contains supplementary material available at 10.1186/s12888-023-05209-6.

## Background

Mental health service use is considerably lower in rural and regional communities than in urban areas in both Australia [1, 2] and internationally [3–5]. Young people (YP) in particular have been reluctant to seek formal mental health support, which has been attributed to stigma and negative beliefs towards mental health services and professionals [6, 7]. Such reluctance is particularly pronounced in rural and regional communities where mental health stigma rapidly spreads through social networks and “sticks” to the individual [3]. This phenomenon has been associated with higher levels of socio-economic disadvantage [8], social conservatism [9] and social visibility in these communities resulting in reduced privacy [10, 11], with lack of confidentiality cited as a major concern of YP when considering accessing mental health services [8]. Professional mental health care in rural and regional areas is often inconsistent and un-coordinated [3, 12], with help-seekers being required to travel large distances [13] and experiencing lengthy service waitlist [14]. The wellbeing of these communities is also more affected by increasingly severe and prevalent natural disasters. Prolonged drought, for example, coupled with feelings of isolation has been shown to impact YP’s mental health, making them feel overwhelmed and worry about their families, friends, money and their futures [15, 16].

While there is substantial research examining the barriers of YP’s mental health help-seeking behaviour in drought-affected rural and regional areas, less is known about what types of supports, programs and education *facilitates* their help-seeking [17]. In a recent systematic review on the barriers, facilitators and interventions for mental health help-seeking behaviours in adolescents, only 19 of 56 studies identified help-seeking facilitators [6]. These included previous positive experience with health services and higher levels of mental health literacy. Most research examining enablers of YP’s help-seeking is from the perspective of adults, parents and teachers (e.g., [14, 18–22]). There is a lack of research examining what YP in rural and regional areas view as facilitators to seeking help when it is needed. The few studies that have examined help-seeking facilitators report that confidentiality and accessibility of mental health services [23], for the availability of more school counselling services [24], and YP desire to develop mental health self-management skills [15] to support the mental health help-seeking process.

This research program had 2 overarching aims. The first aim, which is reported in this article, was to establish a more in-depth understanding of YP’s experiences and needs regarding their mental health help-seeking, and explore mental health education, support and programs in rural and regional communities, complemented by the views of their teachers and parents. The second aim, which is reported elsewhere [25], was to evaluate the batyr@school program which was to be delivered at the participating schools within the year. The schools were selected to participate in the program by the New South Wales Department of Education as they were identified as communities which had, at the time of establishing the research, been experiencing severe drought — a recurring natural event in Australia that has been linked to adverse mental health outcomes in the affected communities [26, 27]. The primary research question for the research reported in this article was: *What are the attitudes and needs of young people, their teachers and parents regarding mental health and help-seeking in drought affected* *rural and regional areas of Australia?*

## Methods

### Study design

This study used qualitative pre- and post-intervention data from an evaluation of the *batyr@school* intervention [28] delivered to YP, teachers and parents in drought affected communities in Australia [25]. Semi-structured interviews (group and individual) were conducted to enable an in-depth exploration of participants’ views, experiences and needs about this sensitive topic [29, 30]. While acknowledging its limitations [31], we used the COnsolidated criteria for REporting Qualitative research checklist (COREQ checklist [32], See Supplementary File).

### Ethics

Ethical approval for this study was provided by the University of Sydney Human Research Ethics Committee (Protocol number 2020/607). Subsequent approval was provided via the NSW State Education Research Applications Process (SERAP Number 2020373).

### Setting, participants and recruitment

The participants comprised three stakeholder groups: high school students aged 14–15 years (grade 9 and 10 in Australia; *n* = 42; 24 females, 18 males), high school staff (primarily teachers but also administrators and Wellbeing Officers) (*n* = 14; 10 females, 4 males) and two parents of high school students (*n* = 2; females). Detailed participant demographics and inclusion criteria are available as a Supplementary File. Participants were recruited through 11 participating high schools who were willing to take part in the qualitative research component of the wider *batyr@school* evaluation. In total, 26 rural and regional school communities from drought-affected areas of New South Wales were invited to participate in focus groups or individual interviews. Focus groups were selected as the main form of data collection as this reduced the administrative burden on schools in supporting the research. Individual interviews were also offered depending on the needs of the participant (eg. time constraints, unable to attend a focus group, personal preference, accessibility issues). Of these schools, 20 participated in the mixed methods evaluation, and 11 consented to conducting the qualitative research component. Of the six schools that did not participate, 5 cited that they were under too much administrative burden to take part, and one school had experienced a recent mental health trauma so felt the timing was not appropriate.

Participants for the focus groups and interviews were recruited through a combination of passive recruitment (via distribution of info sheets through the participating schools’ communication channels to staff, parents and students) [33] and snowball sampling [34]. The intention was to minimise perceived pressure to participate whilst utilising the schools’ social networks [35]. Written informed consent was obtained from all participants.

### Data collection

From June 2021 to June 2022, the chief investigator (a qualitative researcher and psychologist), supported by a postgraduate student, conducted 14 group and three individual interviews with a total of 57 participants. The group interview size ranged from 2 to 5 participants. Of the 11 student interviews, three included the active participation of a teacher. Some schools considered the presence of a teacher necessary because the student interviews were conducted during school hours. Sixteen interviews were held via Zoom, and one parent interview via telephone. The sessions lasted between 20 and 115 min (mean duration 50 min). All interviews were audio-recorded, transcribed verbatim, de-identified and checked for accuracy. One teacher who was unable to participate in an interview provided a one-page written feedback on the interview questions, which was included in the data set.

Two of the research team members, both with extensive experience in qualitative research, reviewed the transcripts alongside data gathering and adapted the interview guide for further interviews as initial themes evolved and the need for more data in particular topic areas emerged [36]. This reflexive iteration between data gathering and analysis also served to refine focus and understanding of the data, and to determine when theme saturation was achieved [37]. The interviews explored 4 questions: 1) participants’ views on how drought impacted their local community from a mental health perspective, 2) participants’ understanding of locally available mental health support resources, 3) YP’s help-seeking experiences including what participants would do if a(nother) YP reached out to them for mental health support, and 4) participants’ views on their communities’ attitudes and beliefs about mental ill health, stigma and help-seeking. Participants were also asked for improvement suggestions in all 4 focus areas. The final interview guide is available as a Supplementary File.

### Analysis

In the process of reviewing the interview transcripts and listening to the audio files, one qualitative researcher developed an extensive analytical memo. This memo initially contained rich descriptions of preliminary themes identified inductively as important relative to the research question, interspersed reflexive comments on their potential meaning and relationships to each other [38]. This expansive phase of memo-writing followed a process of structuring and abstraction of the data in which themes and sub-themes were consolidated in team discussions, using a critical realist approach [39]. As a result of this process, the analytic memo had transformed into a framework that was used to report the results. The authors (who had a diverse mix of backgrounds including: qualitative researchers, mental health professionals, a teacher, a university student and an expert by experience; two of the research team were young people themselves (i.e. aged under 25 years [40]) and one was residing in a regional community) collaboratively refined the written results in several rounds of discussion and editing. The findings were also triangulated with the initial qualitative findings of an internal *batyr*@school baseline evaluation report [25], which had previously been analysed, interpreted and iteratively refined by the same team excluding one qualitative researcher who led this study’s analysis. This collaborative approach to analysis encourages high levels of reflexivity based on comparisons between the multiple personal and professional perspectives of all involved.

### Terminology

This study was set in a high school environment. Therefore, the term ‘student’ is used when referring to the YP who participated in this study and the term ‘young person/people (YP)’ in reference to adolescents generally. The term ‘participants’ is used when referring to all three study participant groups. Otherwise, the participant group is specified; for example, ‘Students said…’.

## Results

In the following results section YP’s perspectives, experiences and needs regarding mental health help-seeking, stigma, and education and support services in drought-affected rural and regional communities are described. This is complemented by the views of their teachers and parents. The participants’ descriptions of the barriers to each of these above areas relating to their community, family, school, peers and individuals are provided. Importantly, these barriers are followed by their suggested solutions and facilitators to help-seeking and support. The most salient participant quotes are included in the text and ancillary quotes illustrating the findings further are presented in a Supplementary File.

### Barriers to mental wellbeing and coping mechanisms

#### Community level

Students vividly described their communities’ existential stressors caused by drought and other recurring natural events such as floods, bushfires and mice plagues, all straining the community’s mental wellbeing (See Supplementary File, Quote 1). In some communities these stressors coincided with high levels of social disadvantage, substance abuse and domestic violence (Quote 2). Participants described how these existential and economic stressors affected YPs’ mental health (Quote 3). Long distances to the nearest healthcare services (GPs, specialists, hospitals), long wait times to obtain an appointment and a lack of local youth mental health services made it difficult for YP and their families to access professional help when they needed it (Quote 4a-4b). Both students and teachers summarised the, at times, complete absence of accessible mental health services and contrasted them to those perceived to be available in urban areas (Quote 5 and below):

*In [capital city], there was a lot of resources; and when I come here, there's nothing […] There's a whole lot of issues with suicide and attempted suicide. I think there's a headspace, but I don't know where that is. I think it might be in [nearest city, two hours’ drive away]. Which is nowhere near here. We're in the middle of nowhere. It seems to me like there's a really high, a lot higher incidence of sexual assault and suicidal ideation here. I mean, it's really hard to compare these two populations, but it does seem that way to me. And there's no services. [Teacher_B4]*

Mental health services that were provided as one-off visits to rural and regional schools were seen as lacking coordination and continuity, impacting their effectiveness (Quote 6). Students also emphasised the absence of suitable places outside of home and school where they could socialise and informally support each other’s mental health. Community events that provided social opportunities were limited, and youth and community centres were perceived as lacking essential infrastructure and personnel (Quote 7–8), and were not viewed as desirable for 14–15-year-olds to attend – being more suited to pre-adolescent children (Quotes 7–8).

#### Family level

With limited access to professional mental health and youth support services in the community, good relationships with parents were pivotal to students’ mental wellbeing (Quote 9). Some students valued talking with their parents about mental health, and felt understood and supported when a parent with lived experience of mental ill health shared their experience with them (Quote 10). However, participants described parents, especially fathers, often as time-poor, absent from home, and/or struggling with their own mental health in the face of the rural challenges (Quotes 11–13). Students (and one parent) highlighted that as parents were often lacking mental health literacy and skills, they tended to ignore or dismiss their children’s mental health concerns (Quote 14–16). This lack of knowledge was attributed to stigma, open conversations about mental health being a relatively new phenomenon, fear of appearing weak, stress, or hoping the issue would just go away (Quote 14–16). Students also reported how, at times, mental health issues entrusted to their parents leaked into the community (Quote 17). Others described how parents acted out their mental health struggles via physical aggression, which was viewed as role-modelling unhelpful behaviours to their children:

*S1: I've seen parents who have engaged in their children's, like, dramas […] And it's so hard, because a lot of the behavioural issues do come from parents. Like, it often stems from like your roots […] S2: And how their parents deal with their own issues, there’s like, a lot of physical fighting in our community too. I definitely notice. [Female Student_B1]*

Some students described situations where they or their friends had felt invalidated by their parents (or carers) when trying to share. YP withdrew from sharing their mental health issues with their parents due to negative experiences with it. This also put up a barrier for some students to access online mental health support in case it was witnessed by their parents (Quotes 18–19). Other YP described that this feeling of reluctance, combined with a lack of local mental health services, made it difficult for YP to seek non-local professional help as they relied on parental support to physically get to an appointment (Quotes 20–22). One teacher also described some parents’ lack of cooperation regarding their adolescent children’s mental health treatment in situations when it was initiated by the school (Quote 23).

#### School level

Students reported strongly valuing the educational mental health programs that had been provided at school by visiting services (e.g., *batyr@school* [an interactive mental health program for high school students in years 9 to 12 delivered by facilitators and young people with lived experience of mental ill health that aims to reduce stigma, and educate and empower students to reach out for help when needed (www.batyr.com.au)]; Tomorrow Man, Tomorrow Woman [a mental health program for year 10 high school students that aims to cultivate awareness among emerging young women and men of the pressures that come from gender stereotypes. The program aims to grow emotional literacy and develops skills around cultivating mental wellbeing for themselves and others (https://leonsec.vic.edu.au/tomorrow-man-tomorrow-woman/)]; Elephant Ed [a sex education workshops for year 5 to year 12 students (www.elephanted.com.au)]), but considered this to be a drop in the ocean. Participants felt that a whole-of-community approach was needed, which extended to including educating parents and other adults in the community (Quote 35 and 35a). Some students had not experienced any school-based mental health programs (noting a *batyr@school* program was scheduled for delivery at their school over the following months as it was part of the research evaluation).

The majority of schools had local school counselling services which provided what was described by students and school staff as essential and sought-after mental health support for students. School counselling, however, was extremely under resourced and not meeting demand — as it was commonly only available one day per week, was often booked out for weeks in advance, and follow up appointments were difficult to obtain (Quote 36 and below):

*We've actually got massive waiting lists at the moment for students to see people with mental health training, counsellors, we've got, we've got an undersupply. We've got a school psychologist who's got waiting lists, we got a school counsellor who's got waiting lists and we've got a student support officer who's got a waiting list. [Teacher_F6]*

A teacher described how their school self-funded a psychologist whose work had markedly improved the mental wellbeing of students over years (Quote 37). As part of this, parents were included in the counselling services to YP and given support how to parent YP with mental health issues which was also viewed as effective approach (Quote 37). Other teachers commented on their school counsellors’ limits and, at times, inadequate qualifications (Quote 38–39). A minority of students were unaware that their school provided a counselling service (“*Do we even have a school counsellor?”* [Male Student_F3]); some students were reluctant to visit their school counsellor out of fear of being singled out by the school community (Quote 40).

Both students and teachers described teachers as essential for YPs’ mental wellbeing. Students trusted their teachers and relied on their ability to help. Teachers were highly committed, and often proud, to provide mental health support to their students (Quotes 42–44). Teachers —particularly those with student wellbeing support briefs in their roles like Physical Education teachers, Wellbeing Officers, Year Advisors and Principals — provided mental health counselling to students when school counselling services were unavailable or when approached by students, both formally and informally (Quotes 45–46). They also provided emergency mental health services for students to support and bridge gaps in the chain of professional and home care (Quotes 47–48), and they supported and educated parents (Quotes 49–50). At the same time, teachers expressed feeling overwhelmed by the extent to which a growing number of students needed acute mental health support. They described this as navigating on the edge between feeling obliged, and wanting, to provide mental health support on one hand and being at their limits on the other. These limits, as reported by teachers, included feeling overstretched and often unable to balance the time to fit mental health education and support in with their multiple teaching responsibilities (Quotes 51–53 and below):

*[Teachers] have got so much on their plate with what they've got to do in learning outcomes and all that sort of stuff. They don't really have time to think about these things [referring to students’ mental health] as well. [Teacher_F11]*

Teachers also emphasised that they sometimes felt ill-equipped regarding their mental health knowledge and skills to help students with complex mental health needs (Quotes 55 and below):

*I feel on edge when I try to give the kids advice because I'm not trained in that, like I know myself, I've raised three kids, you know. I've done heaps in my life experience. But at the end of the day, I'm not a counsellor, I'm not a psychologist […] and yet we’re expected to be. [Teacher_B5]*

Teachers felt uncertain where to draw the line between YPs’ privacy versus their caring responsibility as teachers (Quote 56); and also reported that, despite their best intentions, their own patience and empathy was challenged in regard to students with behavioural disorders:

*We’ll have empathy for kids that have got really obvious issues, but then there's the other sort of kids that’ll be in your class, that exhibit disruptive sort of behaviour, and there might be some sort of issue behind that. And people are much less understanding of that, I mean, I find myself I’m much less understanding of that sort of behaviour than I was previously. [Teacher_B4]*

#### Peer level

Peer friendship and mutual support were of utmost importance to students (Quote 67). One student, for example, enthusiastically spoke of the mental health benefits of having initiated a boys social media chat group for the purpose of mutual support (Quote 68). Concurrently, students were acutely aware of their limitations regarding supporting each other’s mental health. They felt weighted down by the responsibility that came with giving, offering, or even just intending to support their friends, and were extremely keen to learn how to help each other more effectively (Quotes 69–71). A parent described YP’s attempts to help each other as “*the blind leading the blind*” (Quote 72).

#### Individual level

Participants reported above-mentioned existential stressors on the community, stretched and insufficiently equipped parents and teachers, a collective silencing (i.e., not talking about) of mental health issues, and a lack of local mental health support weighed on YP at an individual level. This culminated in students knowing when ‘something’ was wrong with their mental health, but often did not know how to describe let alone deal with ‘it’ effectively (Quotes 58–59). Participants described a variety of unhelpful mental health coping mechanisms that they had used themselves or observed in peers. These included: accepting mental health issues as normal (Quote below) and suppressing negative feelings (Quote 60).

*Our feelings are so normalized that it's OK to be sad a lot of the time. It's OK to be angry all the time, which you don't want to be angry and sad all the time. That's not, it's OK to be sad and angry sometimes, but we don't want to do it all the time. And I think that's another issue. We don't register that something is wrong, you know […] it kind of doesn't process that it's not that's not how it has to be, like you can get help. But I don't think we realize that we need it. [Female Student_B1]*

When the weight of bottling up mental health issues became overwhelming, some YP relieved their mental stress in maladaptive ways, making their social surroundings obscurely yet acutely aware that they were not coping. Such ‘concealed cries for help’ ranged from attention seeking behaviour (Quote 61), physical aggression against teachers (Quote 62), towards each other (Quote 63), and towards themselves in form of self-harm and suicide, with self-harm incidents at times being shared with peers on social media. The latter was described by students and teachers as concealed cries for attention and/or support:

*Suicidal self-harm, which self-harm just seems to periodically go with depression and anxiety, now, that's just how this coping strategy that young people have developed like they don't know what else to do that puts them in this next level. [Teacher_F8]*

YPs’ maladaptive stress relief behaviours further increased the mental stress load on other YP and their social circles. Examples given by participants were students’ and teachers’ anxiety coming to school out of fear of being the target of physical aggression (Quote 64), and getting distressed by seeing peers’ self-harm stories on social media (Quote 65). Posted self-harm stories were also perceived as problematic by some participating YP as they thought to be normalising self-harm as a legitimate means of mental stress relief:

*The whole idea of social media has become kind of destroyed by that image that like to have depression, you've got to be self-harming. [Female Student_B1]*

However, participants also reported YPs’ constructive attempts of dealing with mental distress where parental and professional support were lacking, or unwanted out of concern of being dismissed or singled out. These attempts centred around YP helping (and seeking help from) each other as described in section ‘Peer level’. Several students knew of the availability of online mental health resources from school advertisements (Quote 41). The uptake of these online mental health services, however, was mixed. Some students valued the anonymity that online help offered; others hesitated to use it because of “*the security risk associated with putting stuff out on the internet”* [MS_B3], or because they preferred talking face-to-face with someone (MS_B3). Others hadn’t considered accessing online help at all for themselves (M + FS_F12).

#### Impact of COVID-19 compared to other stressors and major events

While the data gathering for this study was conducted during the height of the COVID pandemic (June 2021 to June 2022), and the impact of the pandemic was touched on in the occasional interview/focus group (mostly prompted by the interviewer), we found that the pandemic did not significantly impact, exacerbate, or otherwise change the nature of young people’s mental health needs. Geographic remoteness, long-term drought, and other natural disasters such as floods and bush fires were together much deeper and complex existential stressors on rural and regional YP and their mental health than the comparatively shorter restrictions to urban areas that came with COVID-19. Social distancing and lockdown restrictions for example were viewed as a non-issue in communities without COVID cases. Moreover, remoteness, social distance and isolation were well-known factors that all participants reported to navigate long before the pandemic started, with its restrictions not incurring a substantial difference to day-to-day life.

### Mental health stigma

In all interviews, participants described the presence of stigma in the community limiting addressing mental health issues and accessing appropriate support, including in the school playground and at home. Mental health self-stigma was characterised by (a fear of) being seen as weird or weak – the latter more so among boys and men; being ashamed to be different; and being (scared of getting) judged negatively (Quotes 24–28). Students and teachers reported how fear-based negative connotations around mental ill health prevented YP and their families from talking about their feelings and seeking mental health support, especially in smaller communities where everyone knew each other (Quotes 29–32 and below):

*They don’t feel comfortable but yeah, or they think oh, that's a shame. I'm not going to be going to ask for help, like, people might tease me. [Female Student_B1]*

Students described how a lack of mental health awareness in the community perpetuated the mental health stigma (Quote 33), and noted that being educated about “awkward” themes made it easier for them to talk about them (Quote 34).

### Mental health solutions

#### Multi-pronged approach to mental health education

All participant groups emphasised the need to take a multi-pronged approach to mental health education and support, with programs and services not only for YP, but also for parents and teachers and the community as a whole (Quote 73-74a), to tackle the communal mental health stigma and enable managing mental health issues without shame (Quote 75). Students in particular stressed the importance of educating parents and teachers (Quote 76). They wanted their parents to gain more mental health awareness, know how to identify mental ill health, act appropriately when their adolescent child displayed signs of mental ill health, know where to get help and how to keep mental health issues confidential (Quotes 77–80). Indeed, students yearned for their parents’ mental health understanding and appropriate behaviour (Quote 81). It was rare for students to report that their parents already had “*a really good idea of what to do*” (FS_F5). Educating parents was also a high priority for teacher and parent participants (Quotes 82–83). All participant groups also stressed the need to upskill teachers on how to identify and support YP with mental health issues (Quotes 84–86). Several teachers wanted mental health education to be part of the core teacher training (Quote 87).

#### Local, face-to-face, free of charge mental health services

Participants wanted mental health services to be offered locally, that is, in their community, to overcome the access barrier and to get personal face-to-face support. They wanted mental health services to be offered free of charge, especially during times of financial hardship caused by recurring natural events (Quotes 88–93). Some participants also emphasised a need to review how school counselling services operate including how well their training addressed the needs of school communities (Quotes 94–95). One teacher considered mental health education as the foundation for other learning to happen and suggested implementing it into the school curriculum (Quote 96).

#### Relatable, engaging, and inspiring mental health programs

Relatability was the desired mental health intervention characteristic that participants referred to most often and most passionately. Specifically, students wanted mental health programs to be tailored to their local circumstances, with demonstrations of empathy and understanding for them (Quote 97). More importantly, participants found programs extremely relatable and effective when they portrayed the perspective of YP with lived mental ill health experience, as in the *batyr*@school program (Quotes 98–99 and below).

*I think it was really good that [Y9 students] heard […] the story of that young girl […] she wasn't going anywhere. She had no money. She was homeless, living in a car […]. But the kids, really, they were captivated by her. I remember looking around when she was speaking and they were just staring at her, like, in awe, yeah. And a lot of kids, when they had to write a message about the program, wrote about her on their piece of paper. [Teacher_F10]*

Students contrasted this with mental health programs that only consisted of standard lecture format presentations, rated these as “*disengaging*” (Quote 100–101). Students also opened up when mental health programs included interactive elements such as group discussions and physical activity games. They found these highly engaging as they could contribute their opinions and experiences, release stress while physically engaging in fun activities, and maintain focus (Quotes 102–104). Group discussions were most accepted when the groups were small and homogenous regarding YPs’ age, gender and mental health needs (Quotes 105–108).

#### Empowering YP

Students highly valued peer friendships, if not depending on them in the face of the rural and regional challenges. They often preferred providing mental health support to each other over approaching their parents or teachers or accessing professional mental health support (Quotes 109–110), especially mental health services with whom they had no pre-existing relationship (see next section). Obtaining practical strategies how to help each other and increasing their confidence in talking about mental health were therefore top priorities for students, rarely addressed by the existing mental health support options available to them (Quotes 111–113). Some of the more outspoken students, and students with a lived experience of mental ill health, also valued opportunities where they could act as mental health ambassadors to give their peers “*that little push to come out*” (MS_B3), pass on their experiences and inspire other YP (Quotes 114–115).

#### Confidentiality and trust

Confidentiality around using mental health support was extremely important to students. Many of them were deeply afraid of being identified as in need of mental health support (due to the stigma identified above); they were only prepared to open up if they felt it was absolutely safe to do so and they could trust their helper (Quotes 116–117). Trust, confidentiality and safety for them developed with knowing the helper, preferably in person, being able to relate to them, and having a positive personal relationship with them. For the students who participated in our study, these criteria tended to be met mostly by peers and teachers, less so by parents and school counselling services, and least by health professionals and online helplines not previously known to students. Face-to-face contact, relatability, engagement and empowerment of YP all contributed to enhancing YP’s trust, as detailed in the earlier parts of this section.

### Early and ongoing preventative mental health education

All participant groups emphasised that mental health education for YP should start early and be taught throughout all high school years, with some participants wanting mental health education to start in primary school (Quotes 118–122). Similar to the mental health education goals for parents and teachers (see section Multi-pronged approach above), participants suggested that mental health programs for YP should raise mental health awareness, teach how to recognise mental ill health, develop mental health self-help strategies, and address and break through stigma and stereotypes that were detrimental to YP’s mental health (Quotes 123–127).

## Discussion

This was the first known Australian qualitative study to explore the barriers, needs and solutions of 14 to 15 year-old YP in drought-affected rural and regional communities regarding MH education, help-seeking and support, specifically focussing on how to facilitate help-seeking. The key findings through the lens of YP, their teachers and parents were that help-seeking would best be enabled by a multi-pronged community approach to mental health education and support. More specifically, they wanted such mental health education and support to be locally available to all community members, free of charge, relatable, engaging, and empowering. Participants felt the need for mental health education to take a preventative approach, emphasise the development of mental health self-management skills, start in early school years, and be offered throughout all high school years to both YP and parents. For teachers in rural and regional areas, facilitating help-seeking for YP with mental health problems led to a tension between being students’ trusted, first point of call for mental health support on one hand and feeling overwhelmed and under-skilled regarding providing this support on the other. Following, a three-dimensional approach to improving the MH situation for YP in rural and regional communities in Australia by facilitating Engagement, Empowerment and Integration is discussed in context with existing literature (See Supplementary File: Principles and Facilitators).

### Engagement

Emotional engagement is crucial in the effectiveness of mental health programs, as for example shown by the literature on contact-based interventions [41, 42]. Our study adds to this literature by demonstrating how *relatability* facilitates emotional engagement and, at least temporarily, this can break through stigma in rural and regional communities and improve YPs’ mental health help-seeking behaviour. Hearing the lived experience mental health stories of other YP, a contact-based intervention and core component of the *batyr*@school program [28], was by YP, their teachers and parents perceived as particularly relatable, engaging and meaningful. Our findings demonstrate how the perceived relatability of this intervention emotionally opened up YP towards sharing their own mental health stories. Given the high acceptance of this type of interventions from this sample, more research needs be conducted into how lived experience storytelling can be harnessed as a tool to make mental health programs relatable, and thus maximise YPs engagement and help-seeking behaviour. Further research into this area could be a valuable contribution to the currently inconclusive literature in the field of contact-based interventions [43].

An important variable in the relatability of lived experience storytelling and, more broadly, a mental health education intervention as a whole, is the delivery mode. While in this study, participants preferred face-to-face over online delivery, citing trust, confidentiality and interactivity as limiting factors of online programs, systematic review evidence suggests that online interventions can provide similarly effective anti-stigma results as face-to-face sessions [44]. More studies are needed to consolidate these findings. In any case, digital delivery has a potential to add extra scaffolding to face-to-face, providing further information and support to those who want it or don’t have access to face-to-face programs, for example, due to their geographical remoteness.

Another variable relating to emotional engagement in this sample of YP was the depth of insight mental health program facilitators had of the local communities they worked with. This included insight into the specific economic and environmental challenges rural and regional communities faced and how these challenges impacted their members. YP for example connected better with counsellors who “*understood drought*” [15] or who had experienced bushfires. Following this rationale, it seems advantageous to deliver locally tailored mental health education and support by professionals who are familiar with the culture and needs of individual communities [10, 24]. Further research into the importance of this aspect is indicated.

### Empowerment

While emotionally engaging YP is important, it is not enough. Corroborating with existing literature, the present study found that YP wanted to feel a sense of empowerment and active participation in the process of learning about and managing their mental health, and seeking help [10, 45, 46]. This sample of YP asked to be equipped with more confidence and skills around talking about and managing their mental health. They described wanting to feel a sense of choice and self-determination. Further, a desire for empowerment was raised by YP alongside their preference for informal, especially peer-based mental health support, which is also a common theme in the literature [11, 45, 47, 48] as is the finding that such informal sources are notoriously under-resourced and under-qualified (e.g., [11]). Further investigation into whether (and how) YPs’ desire for self-determination and peer-based mental health support can be harnessed is warranted. For example, in the context of developing mental health youth ambassadors — as done in the Student Chapter of the *batyr*@school program [28] and the Canadian Stop Now and Plan (SNAP®) Boys (e.g., [49]). This could be especially useful in rural and regional communities where professional mental health services are scarce and YP rely on strong peer friendships. With appropriate professional guidance, there is potential for YP to grow into role models for ‘good’ mental health management, building from the bottom up a positive mental health culture in their social networks including on social media to reshape current, often maladaptive, mental ill health models and discourses [50, 51]. YP’s desire for more educated parents also speaks to a bottom-up empowerment approach to tackling mental health issues in rural and regional communities where parents are often found to feel helpless when it comes to understanding and supporting their children’s mental health [14, 19, 20]. Clearly, empowerment is unlikely to result from one-off interventions. It rather needs a systematic, well-coordinated and integrated mental health approach as outlined following.

### Integration

In line with existing literature, our findings highlight the need for a multi-dimensional integration and coordination approach to the provision of mental health education and support in rural and regional communities. An integrated approach requires first and foremost better coordination between school- and non-school-based mental health services so that YP are offered seamless and ongoing mental health education and support solutions rather than uncoordinated or one-off piecemeal [3, 12]. The current findings also show that embedding mental health education in school curricula earlier, more prominently and consistently throughout schooling years was requested by participants as a way to equip YP with mental health knowledge and management skills before mental health issues arise. While this may not be unique to rural and regional areas, it could be an important avenue to tackling mental health stigma and increasing help-seeking when needed, particularly because these communities can be underpinned by social conservatism and lack anonymity [9–11].

Intensifying school-based mental health education and support, however, has its own issues. Teachers, including welfare officers, have been shown to increasingly navigate at the edge of their time availability and mental health competence, calling for training and resources on how to identify and provide early mental health support for students [18, 21, 52–54]. Recent nation-wide surveys of teachers and the public have shown a link between teachers’ mental health care role and burnout: as teachers assume an increasingly influential role in developing YPs’ social and emotional development, with the public relying on them to do so [55], teacher burnout and exodus of the profession is rising [56]. Furthermore, school counselling services cannot completely meet the burgeoning demands for students’ mental health support in rural and regional Australia. Frequently covering multiple rural and regional communities, school counselling services often operate on a one-day-per week basis, are booked out well in advance and, regularly stretched for time, experience concerning levels of burnout [57, 58]. We recommend more research be done on the role that external/visiting mental health programs can play in rural and regional areas and thus build on selective studies that have shown that such programs can relieve school staff and emotionally engage YP in unique and novel ways that cannot easily be replicated by schools who are bound to rather uniform structures, curricula and lesson formats [25]. There might also be a place for the integration of social workers into a multi-pronged mental health approach with the aim to strengthen social connections and support between YP and their families, schools and communities [59, 60].

Whichever integration model is chosen, attention must be given to the careful coordination of school-based with community-based and visiting services to ensure smooth transitions, consistency and continuation of mental health education and support for YP throughout their formative high school years and beyond [17]. Co-design, also known as participatory design, with key stakeholders including YP, teachers, parents and mental health organisations, could be an important way of ensuring mental health education and support programs meet the needs and wants of (school) communities and the YP they are targeting. Participatory design places stakeholders at the centre of the design process and is part of a paradigm shift towards collaborative bottom-up engagement whereby stakeholders jointly explore and create solutions to program design and service delivery [61]. Recommendations for best practice in clinical mental health service design already emphasise the need to involve YP in the planning, implementation and evaluation of services [62] and this must be extended to community-based interventions [63–65].

### Strengths and limitations

A key strength of this study was the recruitment of a large sample of YP from a diverse range of rural and regional areas of Australia. Their perspectives were complemented and triangulated with those of rural and regional teachers and two parents. However, due to the conservative recruitment methods we used, our sample was likely biased towards participants with a certain familiarity with, and confidence in talking about, mental health issues. Other limitations of this study relating to the participants include the presence of a teacher in 4 out of 7 student interviews, possibly inducing some students to say different things to what they would have shared had they been interviewed without the presence of their teacher. Further to this, three adult participants were interviewed individually, as they needed to be conducted at a time that was convenient to them in order to participate. It is acknowledged that these interviews may glean more information and depth, compared to the focus groups. Further, although we followed processes to establish theme saturation [37], it is acknowledged that only two parents participated, and their views were not drastically distinct to other participating adults. Given the constraints of the evaluation, a greater focus on student recruitment was prioritised, however more diverse set of parent voices could have consolidated our finding on parental support of YP in drought affected rural and regional Australia.

The design of the evaluation research was such that some of the focus groups were conducted prior to the implementation of the *batyr*@school program to address the first research aim (i.e. to understand experiences and needs regarding mental health help-seeking, and explore mental health education, support and programs in rural and regional communities) and some were conducted after the *batyr*@school program was implemented as part of the program evaluation’s second aim. As both sets of focus groups were used in data analysis, results are interpreted through a lens of school-based mental health programs. This is important as although eco-systemic enablers to help-seeking (such as service provision models, family environment, community attitudes) are addressed in the findings and elsewhere in research focusing on rural and regional areas (e.g., [14, 18–22]), the solutions presented in this discussion centre around what schools and the schooling systems can do in practice to enable better mental health education, programs and support. Further to this, topic guide development was influenced by the evaluation questions, not necessarily the literature concerning young people's help-seeking or general mental health and help-seeking when impacted by drought.

Another limitation is that for the focus groups that were conducted after *batyr*@school program implementation, the voices of students who had not attended the *batyr*@school program, and YP outside of the school system were lacking. There is evidence that such unheard voices form a significant portion of YP who need but don’t reach out for mental health support [47, 66, 67]. There is a need for further research into understanding how to engage and support these “silent” adolescent populations to best identify how to facilitate and support help-seeking. Further to this, future studies should consider investigating the needs of young people outside the 14–15 year age range, and seek to compare perspectives based on different demographics including young people who identify with minority groups such as young people with an Aboriginal and Torres Strait Islander background, LGBTQIA + young people, and young people who have a non-English speaking background, particularly those who have arrived as refugees.

## Conclusions

Our study highlights important facilitators of mental health education and support for YP in drought-affected rural and regional areas of Australia. These include a multi-pronged, preventative, local community approach with an emphasis on early and ongoing development of YP’s self-management skills. We propose a three-dimensional *Engagement, Empowerment, Integration* approach which comprises: 1) maximising YP’s emotional engagement; 2) empowering YP to manage their mental health through developing practical coping skills; and, 3) integrating MH education and support programs into existing community and school structures and resources. Integration needs to be well coordinated in respect to time, place and capacity-related factors. Particular regard should be given to the important support roles teachers, school counselling services, social workers and parents can and already do play in the social and emotional development of YP.

### Terms and abbreviations used

YP: Young person or young people. This term is used interchangeably with ‘adolescents’ and refers in this study to 14–15 year-old students who are enrolled in the high school years 9 and 10. The cited literature (Background and Discussion sections) refers to adolescents using a variety of age groups, ranging from age 10 to 19 years (reference [6]), to 14–18 years (reference [7]) and 12–18 years (reference [8]).

### Supplementary Information

**Additional file 1.** 

**Additional file 2.** 

**Additional file 3.** 

**Additional file 4.** 

**Additional file 5.**

## Data Availability

The datasets used and/or analysed during the current study are available from the corresponding author on reasonable request.
