# Supplementary material for: A qualitative exploration of young people’s mental health needs in rural and regional Australia: engagement, empowerment and integration
Source: BMC Psychiatry. 2023 Oct 13;23:745. doi: 10.1186/s12888-023-05209-6 (PMC10571294; doi:10.1186/s12888-023-05209-6)
Supplement: Supplementary file 2 — Additional file 2. [file 12888_2023_5209_MOESM2_ESM.docx]

**Table of Participant demographics**

| **Characteristic** | **Students** | **Parents** | **School staff**  **(Teachers, Wellbeing Officers, Principals, administrators)** |
| --- | --- | --- | --- |
| Gender |  |  |  |
| Female | 24 | 2 | 10 |
| Male | 18 | 0 | 4 |
| Geographic location*ɸ |  |  |  |
| Inner/Outer Regional^ | 7 | 1 | 3 |
| Outer Regional | 20 |  | 5 |
| Outer Regional/ Remote^ | 12 | 1 | 5 |
| Remote | 3 |  | 1 |
| Pre/post intervention interviews |  |  |  |
| Pre intervention | 11 | 1 | 6 |
| Post intervention | 31 | 1 | 8 |
| Totals (n=58) | 42 | 2 | 14 |

* For the purpose of this study ‘**regional’** areas are defined as areas located outside of metropolitan areas with limited access to services. *Inner* regional areas have a population greater than 250,000 people with some restrictions to accessibility to some goods, services and opportunities for social interaction; *outer* regional areas have a population of fewer than 250,000 people and are located further from metropolitan areas than inner regional areas with significantly restricted accessibility to goods, services and opportunities for social interaction. ‘**Remote’** areas are defined as areas that are located far from metropolitan areas with a very small population density and very restricted accessibility to goods, services and opportunities for social interaction as defined by the Australian Statistical Geography Standard (ASGS) Remoteness Area classification and Accessibility/Remoteness Index of Australia.

ɸ Geographic location was determined by location of school, not participants’ residence.

^ These schools were located in mixed classification areas.

**Inclusion criteria**

Inclusion criteria for **participating high schools**: high schools located in drought-affected regional or rural areas of New South Wales, Australia. For the purpose of this study, ‘drought-affected’ communities are defined as areas that are experiencing prolonged dry periods, resulting in reduced rainfall, limited water availability, and a decline in agricultural productivity.

Inclusion criteria for **participants**:

1. Students (grades 9 to 12) who attend a participating high school; or,
2. Parents/ guardians/ family members of students (aged 18 years and over) who attend a
   participating high school; or,
3. Teachers working at a participating high school; or
4. Members of the local community (aged 18 years and over) who are associated with a
   participating high school such as health professionals, other health service providers, and
   other members of the community.
