# Supplementary material for: A qualitative exploration of young people’s mental health needs in rural and regional Australia: engagement, empowerment and integration
Source: BMC Psychiatry. 2023 Oct 13;23:745. doi: 10.1186/s12888-023-05209-6 (PMC10571294; doi:10.1186/s12888-023-05209-6)
Supplement: Supplementary file 3 — Additional file 3. [file 12888_2023_5209_MOESM3_ESM.zip › Supplementary file 3a Interview guide_Baseline.pdf]

Batyr x DoE Service Evaluation

Qualitative Focus Group Guide

Baseline

START

Welcome overview

Revisit Participant Information Sheet

Introduction of participants (1-4 people)

Confirm consent and audio recording

LOCAL COMMUNITY RESOURCES

Explore participant understanding of mental health resources and information for young people in their local area.

Example questions:

- *What resources are available in the local community for young people's mental health that you know of?*
- *What kind of information and resources are provided at schools to support young people's mental health?*
- *Are these sufficient?*
- *How could this be improved?*
- *What kind of resources/ information/ training would you like personally? (Probe from the perspective of participant – student, teacher, parent, community member)*

**HELP SEEKING IN THE LOCAL COMMUNTY**	Explore how a young person can seek help in the local area.<br><br>*Example questions:*<br>• *What are the main people or places for young people who need support for their mental health that you are aware of? (probe: local, internet, phone based support)*<br>• *What assists a young person to seek help for mental ill health in the local community?*<br>• *Is there anything that gets in the way of help seeking?*<br>• *How could this be improved?*<br>• *What would you do if there was a young person close to you that you thought needed help for their mental health?*
**LOCAL COMMUNITY ATTITUDES**	Explore participant perspectives of the local communities views on attitudes and beliefs about mental health, stigma, and help seeking for young people.<br><br>*Example questions:*<br>• *What do you think community attitudes are towards mental ill health in your local area?*<br>• *How does this impact young people who may need support for their mental health?*<br>• *Is there any evidence of stigma in the local community about mental health?*<br>• *Is there anything that you know of that is helping to combat stigma around mental health in your local community such as programs or campaigns? (probe: young people)*

**DROUGHT AND MENTAL HEALTH**	Explore participant views of the local communities experience of drought from a mental health perspective.<br><br>*Example questions:*<br>• *How has the drought impacted the community?*<br>• *Has this had an impact on young people's mental health? If yes, how so? If no, why is that?*<br>• *What do you think is needed to support the mental health of young people in drought affected communities?*
**BATYR PROGRAM**	Explore participant awareness of Batyr.<br><br>*Example questions:*<br>• *What are your views on young people learning about mental health, mental health stigma, and help seeking in school? (Probe: Why is that?)*<br>• *What are your views on young people with their own experience of mental ill health sharing their stories of help-seeking to school students, for example, as part of a school mental health education program?*<br>• *What are your views on the needs of teachers (or parents) to support students with their mental health, mental health stigma, and help seeking in school? (Probe: adequacy of current resources, training, support)*<br>• *Have you heard of batyr? (Probe: what do you know?)*
**END**	Wrap up and thanks
