# Supplementary material for: A qualitative exploration of young people’s mental health needs in rural and regional Australia: engagement, empowerment and integration
Source: BMC Psychiatry. 2023 Oct 13;23:745. doi: 10.1186/s12888-023-05209-6 (PMC10571294; doi:10.1186/s12888-023-05209-6)
Supplement: Supplementary file 3 — Additional file 3. [file 12888_2023_5209_MOESM3_ESM.zip › Supplementary file 3b Interview guide_Follow-up.pdf]

Batyr x DoE Drought Project Service Evaluation

Qualitative Focus Group Guide

Follow-up

**START**	Welcome overview<br>Revisit Participant Information Sheet<br>Introduction of participants (1-4 people)<br>Confirm consent and audio recording
**PROGRAM FEEDBACK**	Explore participant views on the batyr@school program (note: this refers to the student, teacher and parent programs).<br><br>*Example questions:*<br>• *Can you tell me how you found the batyr@school program?*<br>• *How would you describe the impact of the batyr@school program on the school community?*<br>• *What did you like most about the batyr@school program?*<br>• *How could the batyr@school program be improved?*<br>• *How relevant is the program to your community?*<br>• *What are your views on young people with a lived experience of mental ill health purposively sharing their stories of help seeking to school students (as part of a school mental health program)? (Probe: why is that? Has this changed since taking part?)*

**DIGITAL STORIES**		• *[Ask if digital story was presented at the school] How did you find hearing from young people face to face compared to watching a digital story?*<br>• *What are the benefits of digital stories compared to face to face?*<br>• *What are the drawbacks? [If drawbacks are highlighted ask…] how can batyr improve their digital story telling to address this?*
**SROI**	Explore Social Return on Investment	• *CHANGE: Can you describe the main changes (if any) that have come up since the batyr@school program was run?*<br>*Probe: Revisit themes from baseline focus groups including:*<br>o *Help seeking and barriers to help seeking (Probe: impact of these changes)*<br>o *Attitudes towards mental ill health, stigma and help seeking (Probe: personal and school community, impact of these changes)*<br>o *Knowledge of what to do if a young person needed support for their mental health*<br>o *Awareness of resources and information in the community (probe: local, internet, phone based support, campaigns)*<br>o *Changes at school (eg. Training, support, procedures)*<br>• *VALUE: [for each change highlighted ask…] how valuable is this change (Choose appropriate noun/ pronoun: to you/ the school / the students etc.)?*<br>• *DEADWEIGHT: Would some of these changes happened anyway if the program hadn't come to your school? Why?*<br>• *DROP OFF: how long do you expect these changes to last?*<br>• *ATTRIBUTION: how much of the change was because of other people or programs?*<br>• D*ISPLACEMENT: by completing the batyr program, do you think any*

		other activities were stopped? If yes, what is the impact of this?
**END**	Wrap up and thanks	
