# Supplementary material for: A qualitative exploration of young people’s mental health needs in rural and regional Australia: engagement, empowerment and integration
Source: BMC Psychiatry. 2023 Oct 13;23:745. doi: 10.1186/s12888-023-05209-6 (PMC10571294; doi:10.1186/s12888-023-05209-6)
Supplement: Supplementary file 4 — Additional file 4. [file 12888_2023_5209_MOESM4_ESM.docx]

**Table of Participant Quotes**

| **Theme** | **PARTICIPANT**^ | **No** | **Quote** |
| --- | --- | --- | --- |
| YPs’ experiences | | | |
| Community MH support | MALE STUDENT_B3 | 1 | So 2019, when the drought was bad, it was probably the worst thing for mental, like, the worst age for mental illness around this area would probably have been in the adults, the farmers, the workers […] Going through the stress and having no money to feed their animals, to feed their family, and everything went up through those years of drought. [...] Now that we're out of [the draught] and we've got massive flooding coming through, it’s ruining all the farmers’ seeds and stuff. They get out of their crops and they're ruining all the hay going through all that. And then once the mouse and rats have been through it, it's no longer sellable. So then obviously they're going to be stressing off that, feeling bad off that, thinking thoughts that people don't want to think. |
|  | TEACHER_B5 | 2 | The vast majority of our clientele within the school are […] dependent on social security […] drugs are pretty predominant in the community […] 15 years ago there was a lot more alcohol, now drug dependency within the family is pretty predominant, and that's seen as just the norm. And we have a pretty strong indigenous community. And the traditional structures that they have as a community has really depleted as well. The reliance or even respect for elders is just gone out the door in the last generation […]. That brings obviously with it other things like domestic violence and the kids being neglected […] there might be one or two times where the police have actually done a drug raid or actually done anything about the drugs coming in and out of town. It's a free ride for them, so law enforcement we have a presence but we really don't have any influence on that side of the law. |
|  | TEACHER_B4 | 3 | I remember that time at the school and like [the bushfires] just went on and on and you could really feel it in the school grounds with the kids. You know, like some students would come to school so upset because they've had to sell their horse that had for years or they had to get rid of their calves or, you know, and like that sort of thing. So there was that spill over […] it's not that they will talk about that, but you could see that was, like they feel bad about not being on the farm, helping mum and dad […] that impacted definitely on their education and just their motivation […] They were stressed. |
|  | TEACHER_B4 | 4a | T1: one of our girls who attempted suicide 18 months ago now, they transferred her to I think there was a unit in Newcastle. So how far away is Newcastle? […] T2: Six or seven hours away T1: And she was there for many weeks, so her family had to like, have either someone down that way or paid for where they stopped |
|  | WELLBEING OFFICER_F8 | 4b | There was a young person that was admitted into the hospital […] she self-presented, waited six hours in the [Emergency] waiting room after taking two packets of Panadol, went and then was sent home before being assessed by anyone in the mental health team of the hospital, so they just assessed her for physical like from side effects, but not a mental health assessment at all. Even though she was presenting from an unsuccessful suicide attempt […] unless you're over eighteen, there isn't really a place and there isn't an equivalent adolescent mental health clinic […] if these young people are only being sent home, they then potentially really hurt themselves at home. I just don't know how we get off that cycle and we move any forward to a greater outcome. I just think if you broke your leg, you'd be in the hospital waiting to have surgery within hours. But then if you are saying you want to die and you want to go to all lengths to kill yourself. What's that what's the what's the answer? It's just I just it's really sad […] Especially if you're under 16. |
|  | TEACHER_B5 | 5 | There is really a lack of services. There's not much in the town. You do have to travel, and some people don't have the means to travel. |
|  | TEACHER_B5 | 6 | There's very little effective support coming into these town […] They're just so understaffed […] they haven't got the time to give them an effective service […] they haven't got the initiative or the drive or the organizational skills to be able to run that […] there's no accountability and no one knows who is servicing what town. […] So in that six years we've only had four and probably three to four of those, one was okay, the other two, three, have been variant effect […] That discontinuation, that discontinuity… |
|  | MALE STUDENTS_B3 | 7 | S2: there's not really anything out here that people can go to. S1: That's why I mentioned like a cafe because then that's not a professional organization where people are being embarrassed to go into but allows that window for people to talk about their lives outside of the normal school life […] the young kids are not going to go to the bar to talk to their friends S2: There's not really a practice of socializing here outside of school and the pub. Other than that, you're on your farm or you're in town like doing your shop |
|  | MALE STUDENTS_B2 | 8 | MS1: there's a youth centre in town [I: Do you think it is used very much?] S1: Not really [I: How come?] S1: There’s not really any worker, I think. S2: Yeah, no one really runs it. [I: Are there any other places for young people to go in town?] MS2: Not really, no [I: You also mentioned a community centre. Is that nearby?] S1: Yeah […] S2: There's people there, you walk in, there's games, basketball […] S1: Yeah, tennis court by the shed […] S1: It’s kind of for little kids [I: So, it’s not for you guys, the kids your age?] S1: Not really for older ones. Yeah, not really. [I: Where do the older kids hang out?] S2: […] nowhere, just stay at home. |
| Role of parents | FEMALE STUDENT_B3 | 9 | Can you imagine if there was a kid who didn't have a good relationship with their parents, with the fact that it's possibly like hundreds of them, and their only escape was school and the fact that there's nothing else out here for them? |
|  | FEMALE STUDENT_B1 | 10 | I predominantly have one parent in my life and she has experienced mental health-related illness herself. And that's been good because she's been able to, I don't know, be real with me, like you know about those things and be really supportive. |
|  | MALE STUDENT_B3 | 11 | Especially in these rural communities, a lot of people are busy like the parents say the son is either, your brother is either working with the dad on the fences or trying to relax with his mates, and there's not a lot of talking that you can do, in amongst chores and work. |
|  | TEACHER_B5 | 12 | We have not many dads who would have any sort of role let alone an active role in the upbringing of their kids. |
|  | PARENT_F9 | 13 | Often, a lot of parents suffer those social, emotional health issues themselves but don't recognize it. |
|  | FEMALE STUDENT_F3 | 14 | I think because it's mental health, like, just talking about mental health is so new. I find, a lot of adults they find the concept hard to grasp about like going to talk, go talking to someone about it, about their feelings. |
|  | FEMALE STUDENT_B1 | 15 | Parents feel like they can't share that with their kids, so like don't want to discuss it if they if they're having a hard time, they're not going to want to discuss that sort of thing with their child […] I think that's the stigma, I think, like the stigma surrounding weakness |
|  | PARENT_F9 | 16 | It doesn't matter what age [their kids] are, parents battle with knowing what to do and how to support their kids when it comes to social and emotional health […] Parents are making not so good decisions because they're stressed or worried about their children, so they're pestering or ignoring or go away. |
|  | MALE STUDENT_F1 | 17 | Sometimes you speak to one parent, they tell the other parent, but then they both can't find out something, they will probably go their own separate ways to get outside of help, but then stuff about the chain reaction until you find someone |
|  | FEMALE STUDENT_B1 | 18 | I personally don't live with my parents, I live with my aunt, […] I don't talk to her about how I feel because she brushes it off and I see that very, very largely in my personal friend group and just even people outside of my friend group I know that they aren't talking to their parents. |
|  | MALE STUDENT_F1 | 19 | if you're at home, you know if you've got problems with your parents, you don't want them to be able to overhear [accessing online MH support from home]. |
|  | TEACHER_B4 | 20 | I don't understand how even if the kid, even if there was a sexual assault counsellor, how is the kid going to actually travel […] unless they're going to, I don't know, find a sympathetic parent or someone to drive them […] these kids can't go anywhere unless someone drives them […] Parents’ support is key here because nothing is close. |
|  | TEACHER_B5 | 21 | A lot of these [young] people would not have the means to get, literally would not be able to have someone to drive them down there. |
|  | FEMALE + MALE STUDENT_F12 | 22 | [I: I guess [a one-hour drive to the next MH service] means that some people who are wanting to seek help have to have a conversation with their parents around getting there?] FS: Yeah, yeah, and they could find that embarrassing [I: so that’s a barrier?] MS: Yeah |
|  | TEACHER_B4 | 23 | I know that despite some students having had serious mental health issues, the family will […] forbid medication. They have this fear with, you know, and these are the quite Christian families, they will not. It's like, No, no medication. You just deal with it the best you can. This is sort of your lot. So, you know, we can deal with it as a family. I know a couple of people like a student and that here that where that has happened and it's been serious mental health issues such as, you know, suicide, attempted suicide, requiring hospitalization for a long time, you know, and no medication like they are not willing to. It's like a stigma, like if you need to go on the medication or, you know, you're crazy or you're really bad. So they don't go that step […]I drew up a sort of plan for this girl that had anxiety and one of the things that I put on it was that she should do the Brave program. And, you know, when I checked in with her, they hadn't bothered to do it. And yet the mother was really insistent that the school had to fix her daughter's anxiety. And yet I gave all these suggestions and put all these things in place. And the thing that was going to help her the most, they didn't bother doing. |
| MH stigma | MALE STUDENT_F3 | 24 | We don't get to talk about it as much and we'll feel like, well, we would just get shut down and it would be like […] you should man up and what not with the boys. |
|  | MALE + FEMALE STUDENT_F5 | 25 | [I: Why do you think that people might not want to open up?] S1: They are embarrassed. S2: Just society might judge them. |
|  | TEACHER_B5 | 26 | [YP] don’t feel comfortable but yeah, or they think, Oh, that's shame. I'm not going to be going to ask for help, like, people might tease me or something. |
|  | FEMALE STUDENT_B1 | 27 | Other [students] then tend to belittle their experiences because you know, someone else like kind of poke fun at them a little bit by doing it, you know |
|  | MALE STUDENT_B2 | 28 | [I: If you found out that one of your mates or their parents had a mental health issue, what do you think, people would think?] They're a bit weird. |
|  | MALE + FEMALE STUDENT_F4 | 29 | [I: Why do you think some YP don’t want to find help?] S1: Scared S2: Yeah, like of embarrassment […] And also because she's a really happy person at school, like really happy person at school. And you wouldn't really think she's dealing with those problems of hers, but, yeah, I think that's also what she didn't want to, why she didn't want to seek help because she didn't want anyone being different of her that, like she wasn't that happy person that she makes it out to be. |
|  | TEACHER + MALE STUDENT_F5 | 30 | : We live in a very small rural community and I think gossip is rampant […] Everyone knows everybody and everything about them. S: Got to watch what you say. |
|  | FEMALE STUDENT_F3 | 31 | I think it's the stigma around mental health. Everyone thinks it's such a bad thing and everyone's very reserved about their own mental health and how to act on it, yeah. |
|  | TEACHER_F10 | 32 | You still talk to someone my dad's age who's in their 60s about depression, and they'll say, No, you don't talk about that, you know, mother's post-natal depression, No, that's not real. It doesn't happen. […] there's a big blanket over mental health because people think you said the word mental health, they immediately think negative mental health. |
|  | MALE + FEMALE STUDENT_F12 | 33 | MS1: I don't know anyone with mental illnesses or when we hear about it around town that could be because people are hiding it due to lack of awareness […] FS: I think people are very ignorant. They have ignorance when it comes to this sort of stuff because of [inaud]. [I: do you think it's because of lack of lack of awareness?] FS: Maybe, yeah. MS2: Yeah, probably. Most likely. |
|  | FEMALE STUDENT_B1 | 34 | The STDs and stuff, like it was more, it was more of a conversation type thing because you can get that big health talk and stuff and it's really awkward kids are laughing. Whereas this was, I think we actually got real education about it and kind of normalized that with the kids who might not receive that at home |
| School-based MH education programs | MALE STUDENT_B3 | 35 | [Tomorrow Man, Tomorrow Woman] came to school, it was a half day type of thing. So the men and the women both split up and go into separate thing. So it's a bit more private because the women can talk between women and like men can talk between men. So it's probably a good thing to do as well. If it would happen more regularly, I feel as though people would be able to talk and people wouldn't be bottling up as much as they are. Like in my class they got a lot of the boys talking that you never normally hear talk about stuff. |
|  | FEMALE STUDENT_B3 | 35a | Other than Tomorrow Man and Tomorrow Women, there isn't really anything because it's more of a recent thing, these programs. Like, I never had any of this when I was in younger, secondary and primary. And even if it does come out, it's at the school and it's the students only. And like adults, they don't have any of the programs, the only actual people advertising it outside of TV ads like an ad you might scroll past on your phone, there's like no faces for an adult to talk to. |
| School counsellors | FEMALE + MALE STUDENT_B3 | 36 | S1: Having her here once a week, she's always so busy because so many people are going to try and see her. And sometimes it's like three or four weeks before we actually get to visit […] S2: And when [counsellor] is here for one or maybe two days a week, but that is if she is here, but then if somebody is upset and feeling real down and then they got actually somebody that is talking to her at that time about mental health, they could then get put in and then the other person gets pushed back to later. And then by that time, [counsellor] is already gone or she can't talk to the people that she actually needed to come talk to […] S1: it's like you're fighting for like someone to talk to, sometimes. |
|  | TEACHER_B5 | 37 | One of the best things we did for this school is we hired a counsellor, a psychologist in the school. She comes up any one day a week, and she's here four and a half years now I think at least, yeah so she's part of the school and you'd agree, it's been a phenomenal help for the school, like it started off, like we had a lot of kids right off their tree, just really battling how to actually like, and we've got none of those any more. So she's really helped to settle that. Settle those types of kids then she, a few years ago, then she started working with the SRC, doing group work in classes. And one and another aspect of that also has been obviously a lot of their issues with kids coming in from the door, through the door. So whether it's trauma that's going on at home, or just the parent's lack of knowledge how to parent, basically. And so there's been a fair bit of, by herself still, without tricking them, she'll ring the parents and say look I'm about to talk to your child, do you mind coming in and giving me a bit of background. So yeah, the parents, they really do come in because I'm having a battle with it, and so she'll say, ‘I wonder if I can talk with you a bit just so you can help me out’. But still, she's actually teaching them how to parent and help both of them to stick together or work together as a family unit |
|  | TEACHER_B4 | 38 | There is also a need for specialist sexual assault counselling and school counsellors aren't really equipped to deal with that |
|  | TEACHER_B5 | 39 | So the bottom line is ineffective classroom teachers hop over the fence and become ineffective counsellors. They haven't done, they didn't have the skill sets to start with. |
|  | FEMALE + MALE STUDENT_B2 | 40 | [I: What are your views of people seeking help from a school counsellor?] S1: Some people, like, might not open up. S2: Yeah, like they don't want to talk about it. [I: What makes you say that?] S3: You're scared, people might find out. |
| Online MH resources | MALE + FEMALE STUDENT_B3 | 41 | S1: there's always access to other places online, like Kids Helpline, Lifeline, Headspace, Reach Out […] S2: We have these places that are around actually the big things and links to certain websites and phone numbers around the school. [I: Do you think everybody in the school, obviously, you guys are aware of that. Do you think the other students noticed it?] S3: Yeah. Yeah. And like sometimes there are talks that are, and I think are going around the start of this year, we had like a little card thing that was handed out to us with these on it as well. |
| Role of teachers | FEMALE STUDENT_F3 | 42 | I know a lot of the girls, they hold a lot of trust in some of our teachers and now they'll go to them whenever they're feeling down or anything that's happened. |
|  | TEACHER_B5 | 43 | We are in a good place to, you know, whatever we put in front of them, they generally take on board wholeheartedly. |
|  | TEACHER_B4 | 44 | We're here every day. They [students] disclose. |
|  | FEMALE STUDENT_B3 | 45 | We've got, like, teachers who are advisors for students that we can talk to, like, if the counsellor’s not there. Because the counsellor is only at school one day. |
|  | TEACHER_F10 | 46 | I've got a pretty good relationship with most of my kids, […] most of them can comfortably come to me. Even the boys, actually. […] I wouldn't be surprised if a couple of kids came to me next week with, even if it's just something little, like, ‘I'm having trouble sleeping, what could be wrong with me?’ kind of thing […] the thought process is there for them |
|  | TEACHER_B5 | 47 | if we've got a kid who is self-harm or suicidal, there is, there is, and it's a system […] we're supposed to be able to take them to the hospital |
|  | TEACHER_F8 | 48 | If you present with, ‘Oh I've overdosed on’, and this is from real experiences with my young people, is it, you've just on the weekend taken two packets of Panadol or whatever. And your success is obviously unsuccessful. And then you go through the hospital, […] you go home, like the CAMS goes, ‘We'll be in touch’. CAMS have one and a half staff. So then it becomes, so then my work is actually just around safety, how do I ensure the young people's safety. So, so many young people now have exit cards to me, so I can just physically keep an eye on them. And I've had to bring parents and ask them, can they change their work hours now to ensure that they are home by 3:30 so I can adequately send them from school to home so that I know that there's going to be another adult with eyes on them. |
|  | PARENT_F9 | 49 | I think schools play a really big part in identifying and supporting parents to give them what they need as in there is this headspace, or you can go to these, so you can go to that, or there's online, you know, where you're being on the phone and you can talk if you can't access someone face to face |
|  | TEACHER_F10 | 50 | I've actually thought about starting or doing a mental health information night for parents at the local bowling club. Once a term, I was going to focus on something, something different each term or even in the school hall, just so that parents can understand some of the things that do go on but that they're not necessarily aware of. So the mental health problems that come from social media, um, what is mental health, like, why is your kid going home from school locking themselves in their room? Why are they spending an hour in the shower? You know. Why are they cutting themselves? Why are they thinking of suicide when nothing is wrong or nothing you know of is wrong? I just want to get a bit more kind of like information out there. |
|  | TEACHER_F10 | 51 | I can give it [MH support] to them [students] if they come and ask me for it, but it's not my job to feed out that information. |
|  | TEACHER_F10 | 52 | And as much stress as [being a year advisor] does bring, I feel like I have no time to even scratch my own head. |
|  | TEACHER_F11 | 53 | I'm a PE teacher, so we touch on mental health but really, it's probably not in the depth that it should be. It sometimes can be, because we've got so much content to get through, it's like, ‘Alright, this is what resilience is. This is’, and it's probably not done to the quality sometimes that mental health could be done. |
|  |  | 54 |  |
|  | TEACHER_F11 | 55 | As a young teacher, especially like I definitely felt that I wasn't equipped for a lot of situations. I mean, I still feel like I've got a lot of room to grow in just being able to approach some of those yeah situations regarding someone's mental health and things like that, or just reading the situation a little bit better because like a classroom setting is definitely, can be diverse, let's put it that way, but it's easy for that middle range student who might be not traveling the best to sort of fly under the radar a little bit […] but I definitely feel now, though, with that PD that we did, I have a few strategies to sort of move forward and sort of, yeah, have a, be someone that I can talk to or listen, like yeah listen to them. […] Does modern schools or curriculum really hit what we need to be hitting in regards to mental health? The answer is probably No. No one really feels they know how to deal with it. |
|  | TEACHER_B4 | 56 | T1: for us sometimes it really does take you by surprise, like what some students do go through […] a lot of teachers really don't know what's going on. Like outside of school, even sometimes within school, because of confidentiality and whatnot […] T2: Yeah, that's the biggest challenge for me, like, I mean sometimes you have yeah, might have the basic knowledge, but you really don't know what's going on. And it can be hard because you might sort of stir the situation up without meaning to, the girl who was sort of struggling from, sort of the eating disorders, I had her the morning before she really was struggling and sort of went home, I mentioned that we were having hot chips. And I don't know if that like sort of set her into a bit of a spiral or not, so I was thinking about just driving her home, but it's just little stuff like that where, yeah, you don't really know, and I don't think I don't know what the actual confidentiality sort of stuff is. I don't think we sort of necessarily are allowed to know too much, but it can be hard to sort of, I mean, we've got, we've been equipped with these skills to help the students, but it's a bit hard when we don't know sometimes when we need to be helping them. |
|  |  | 57 |  |
| YPs’ coping mechanisms | FEMALE + MALE STUDENT_B3 | 58 | S1: they've got to listen in on their parents stressing about it so they can hear from their parents. Even if they're not following the conversation, they can feel that something's wrong. And then if their friends and families are affected by it, like with the fires and the floods, like people couldn't get to school and you'd have people's friends at school stressing about their friends who aren’t at school S2: or their families or homes are close to the flood that come through, and they're worried about their family's safety and whatnot […] S3: And what we were saying about, say, if the farmers, they lose all their produce and obviously they'll be starting to get stressed out […] their parents might be a bit angry, a bit agitated and could possibly take that out on them. But it could be a factor too […] because I've seen some kids come to school upset, you’re trying to do the best you can to look after them. But there's not really much you can do. S1: And then you get financial trouble, you don't have the money to go anywhere to get help for your mental health, because this is either way you've got to pay for petrol and you've got to get that referral to the mental health and sometimes you have to pay and there's just not enough money for that. |
|  | TEACHER_B4 | 59 | A lot of the things that we do in pastoral care are actually getting them to choose the topics. And they all want they all want to focus on toxic relationships and those sort of things. And then then, you know, a kid will get really upset and run out of the room, and that's how they come to disclose what what's going on for them. |
|  | MALE + FEMALE STUDENT_B2 | 60 | [I: The mice plague, that's pretty bad out there?] FS1: Yeah, it was horrible […] MS2: Oh, the smell is really bad [I: That's hard on the farmers, isn't it?] S (group): Yeah. FS3: Even the local supermarket. They've had to take everything off the shelf […] S2: And all the farmers maize, it's all got eaten. [I: And how are you guys handling that kind of situation?] MS4: Easy, for us, ‘cause we're not farmers. […] [I: So has it had an impact on your mental health, these kind of events?] S2: Not really […] [I: How about anyone else in the community, parents, adults, teachers?] S1: Well, our parents they would have been angry, they had to clean everything up. S2: Like bait them […] the supermarket closed the door, you couldn't buy anything. S4: It was all sold out. [I: what's your opinion on what's needed? What kind of support is needed?] S2: A lot of money S4: Yeah, and just support. |
|  | FEMALE STUDENT_B1 | 61 | There's also a thing of, I don't know how to word this. There is a need for especially in younger year groups to do things, seeking attention from people, and I understand obviously that could be a cry for help in itself. |
|  | TEACHER_B4 | 62 | There’s the other sort of kids that’ll be in your class that exhibit disruptive sort of behaviour, and there might be some sort of issue behind that. |
|  | FEMALE STUDENT_B1 | 63 | [I: Is there anything else that's sort of happening in relation to mental health and wellbeing at the school that you think is important to flag?] S1: The fights. The fights are pretty bad like physical ones. It's now become a standard that if you have an issue with someone at our school, you take it out physically. There is no intervene, like there is obviously teachers just try to intervene, but between students there's not really much intervention and actual communication. A lot of it's about nothing, like, you know, ‘I heard her call me bitch. Now I'm going to go fight her’ type thing. […] S2: I think there is more female fights than there is male fights to be honest. […] S1: We would have, you know, violence every day. S2 Yeah, there was incidents where it was for every day. S1: Yeah. And that’s kind of coming back a little bit |
|  | FEMALE STUDENT_B1 | 64 | S1: I know personally I struggle with the anxiety of coming to school knowing that there was that risk of like being hurt or maybe someone trying to engage with the physical fight with me. […] S2: I think [teachers] would have to feel anxious coming to their workplace knowing that stuff like this happens so regularly. |
|  | FEMALE STUDENT_B1 | 65 | I definitely have seen [self-harm postings on social media] from the year 9s. And it does worry me a lot, like I don't feel comfortable with that. But it's definitely not something to come out and say to someone like, I'm not comfortable with you doing that. So I think it puts a lot of people in an uncomfortable position. |
|  | FEMALE STUDENT_B1 | 66 | People posting things like [self-harm] on their [social media] story, like, can be quite like triggering […] it’s triggering other people to do those behaviours’ […] if you see so many people posting it, then you think, ‘Well, now I've got to take out my horn that way’ […] S2: the whole idea of social media has become kind of destroyed by that image that like to have depression, you've got to be self-harming |
|  | PARENT_F9 | 67 | My daughter, who like doesn't display any mental health issues but supports a friend that does, and […] her friend won't tell her parents, but she offloads on to my daughter. |
|  | MALE STUDENT_F3 | 68 | [The boys chat group is] probably one of the best things that happened in our lives, honestly, because like instead of being closed off and being, I don't know, like somewhat being two faced in some sense, like it gives us a safe place in some sense where we can open up and where we can talk about our feelings without getting shut down |
|  | MALE + FEMALE STUDENT_F2 | 69 | S1: As friends, we want to help our own friends […] S2: Your friends might not want to go see someone that's the hardest bit because then you don't know how to help them, but you try and do what you can, but like, they can push you away sometimes. |
|  | PARENT_F9 | 70 | [My daughter] feels she has a lot of weight on her shoulders knowing her friend’s secrets, where her friend won't tell her parents. |
|  | FEMALE + MALE STUDENT_F4 | 71 | [I: Can you tell me a little bit more about what you mean by being scary to help someone out?] S1: Well because, like, you might not know what to say. You might like get scared of saying something or like you might offend them in some type of way like, maybe and that also might scare you and especially if it's like a friend and if you say something wrong, like you might like feel like you're going to lose that friend or something. […] S2: Yeah, it’s a touchy subject. S1: Yeah, and you also want like you might not know, you might just like, get awkward or something. |
|  | PARENT_F9 | 72 | They're only young, they don't know what to do, they don't have the life experiences that we have. That's the blind leading the blind really. |
| YPS’ solutions | | | |
| Multi-pronged approach | FEMALE STUDENT_F3 | 73 | Do you just go around to schools or like would you have a program like run in like a town say and have everyone able to attend in the community? […] I think the community would be nice to have that as well, not just as kids […] Why don't you guys target adults more? Like, why are they less targeted in some sense? |
|  | TEACHER_F11 | 74 | We've got to really touch the broader community because of COVID this year. Like we're going to have a big wellbeing day at school and do some things out in the community and involve different businesses and stuff like that. But that obviously was stopped. But if that could happen, I feel like that would definitely assist in sort of, yeah, going back to the homes and the local community as well |
|  | FEMALE STUDENT_B3 | 75 | Just letting people know that it’s okay to ask for help. |
|  | FEMALE STUDENT_F12 | 76 | [I: Is there anything else that you’d like batyr to be doing?] FS: I think maybe if they went and visited like small towns and that […] and then to like parents and like teachers, anyone could go and see. |
|  | MALE STUDENT_F4 | 77 | a lot of the parents don't know much about mental health awareness, like a lot of people. I thought it would be good to teach them just as much as the, students or kids |
|  | FEMALE STUDENT_B1 | 78 | I think that maybe educating parents about what mental illness within their children might look like because what they perceive it as, might not be, what the up to date definitions of it are and looking for those signs that your child could be self-harming or doing those things to make sure that they can intervene because like as teachers and people surrounding them that aren't directly in their household, it can be hard to do that, especially given that you don't see the child on an everyday basis. You can't always check in with that. And there are protocols to say that you can't invade someone's privacy, whereas parents have more control over what they see and how much they are in a child's life and can help to steer them in the right direction and get support. |
|  | TEACHER + FEMALE STUDENT_F1 | 79 | T: Could they do parent stuff? S1: Parent workshop […] S2: Even like resources to parents like, Ring up Mr [xxx] like if your child needs information about what to do and like signs look out for. S1: I feel like it's more helpful for parents as well. |
|  | MALE + FEMALE STUDENT_F1 | 80 | MS: If we can educate parents as well to know the information FS: And to know the answers MS: Not the answers, but like know some facts and that, that we can, they can do we don't have to bring out the outside resources to help […] FS: It's not even about help though, it's just about like mom likes some gossip, and then, Dad will know, and then grandma will know and my Aunty will know, her husband will know, my cousins will know, and it just goes on. |
|  | MALE + FEMALE STUDENT_F4 | 81 | [I: What are your thoughts on reaching out to the adult population?] MS: It would be good because they go through problems too and they can help like their kids and younger people who go through problems too. FS: Yeah, like they would have more of an understanding of mental health. Because in this one story that we heard from the girl from Batyr, she actually said that her mom didn't really believe, well not believe her, but like, didn't really think there was anything was wrong with her. So. Yeah, I reckon if it was like online back when she was a kid, maybe her mum would have been more aware. And actually would have taken her to get that help quicker. |
|  | WELLBEING OFFICER_F8 | 82 | Educating our parents is a big part of the big problem. Like, the whole problem. |
|  | PARENT_F9 | 83 | I think it's just empowering parents to really look for the signs, look for things, listen to their kids and really listen to what they are saying, if it's a cry for help. Or if they come to you for advice, if there's something else going on, maybe not with them, but with someone else that they're trying to support’ and F9 36:46 ‘I suppose at primary school you very supported by having very supportive teachers and like you're still very orientated with your family, you don't go off to work or you don't go and do sport or you don't spend lots of time on your own with your friends. And then when you hit those teenage years, parents are sort of side swiped or sort of blinded by the changes that lots of things occur like at puberty, like, lots of things come into play and you don't have that control you had when you're in primary school, like, they tend to grow up very quickly and start to become more independent. And you don't have that, your finger on the pulse as much […] and as a parent, preparing yourself. |
|  | TEACHER_B4 | 84 | It's the training and I think that needs to be core if we're going to get young people to join the [teaching] profession and prepare them and give them a few more tools to feel confident […] they should be looking at preparing teachers for the mental health issues that our society has today for the social issues and not just looking at an American text on adolescent development and that's it […] and changing attitudes in teachers because some teachers just see it, ‘that's a good kid and that's a bad kid. And they still think like that. |
|  | TEACHER_B1 | 85 | [I: What would there be to support teachers in relation to those challenges for the students’ mental health?] I think education, probably more so about how to, you know, deal with the kid who is being defiant without having to go to the point of, you know, breaching protocol and stuff like that. |
|  | FEMALE STUDENT_F12 | 86 | [I: Do you think it's important for the teachers to see these kind of MH programs?] Yeah, because I think that like it's like they can have like new ways to, like, get around it instead of being just upfront and like, 'Oh yeah, do you have like, do you need help?', or whatever. [I: So they know to sort of ask the questions in the right way about [inaud] thing the students’ needs that?] Yeah. So there's like other ways to go about mental illness, not just like one way. |
|  | TEACHER_B4 | 87 | I did some workshops like The Accidental Counsellor that my school sent me off to in [city] because I was doing that role, Year advisor and stuff. I know our school we've got this segment, Mental Health First aid, Youth Mental Health First Aid. Two-day course […] And I said this absolutely needs to be part of the core because I end up just finding it as an elective and I said no this needs to be part of the core eight or nine units that you guys are doing and they need to be and the service needs to be longer. You can't just do four weeks, four lots of four weeks and then jump in. |
| Local, face-to-face, free of charge MH services | FEMALE STUDENT_B3 | 88 | And then you get financial trouble, you don't have the money to go anywhere to get help for your mental health, because this is either way you've got to pay for petrol and you've got to get that referral to the mental health and sometimes you have to pay and there's just not enough money for that […] There's this place I had to even pay for it to get in and like it should be, shouldn't have to pay for it. |
|  | MALE STUDENT_B3 | 89 | There should be a support system out here in place, because as I said before, it's not just the people around our age, it's the people who are younger, it's also the people that are much older that are the ones that are affected by it. So getting those types of resources and a place out here that people could go to […] they don't have any of the programs, the only actual people advertising it outside of TV are like an ad you might scroll past on your phone, there's like no faces for an adult to talk to |
|  | FEMALE STUDENT_B1 | 90 | And actually getting readily available access to services, like some kids can't make the commute to Headspace because their parents might not have a car and you know, they're not prepared to ask someone else for that, because it's quite an uncomfortable conversation. So we need those assets here, like we need it at school where they come, you know |
|  | MALE STUDENT_F3 | 91 | I reckon they could even, I know, it would be hard and be a lot of money involved in it, but get like a small little shopping down the street or somewhere where you've got workers out here where they can, where the members of the community can go in and then then talk to them and find that bond with them, I suppose. |
|  | TEACHER_B5 | 92 | if the community knew that there was a door, they could knock on and say, I need help, I'm not coping. Maybe. […], if they knew that there was someone that was reliable and consistent and trustworthy there to support them, they would use it.’ B5 54:29 ‘I think even just a check in on the kids that, everyday kid, you know like, just a little group or something like that because you never know’ |
|  | TEACHER_B4 | 93 | that would be my first thing to have someone on the ground, now in the school every day |
|  | WELLBEING OFFICER_F11 | 94 | there was like a few teachers here, if I had to go and take a student out of class and have a conversation with them, that would be like a little bit hesitant or, No, sorry. You need to come back, sort of thing, like it's just so class and school work focused. |
|  | TEACHER_B4 | 95 | There is also a need for specialist sexual assault counselling and school counsellors aren't really equipped to deal with that. And I don't know. I don't know how kids around here get access to that. There's a lot of them that have got those issues that they're just dealing with on their own. |
|  | TEACHER_F11 | 96 | The school doesn't really have wellbeing lessons […] schools have this big this big focus on numeracy and literacy and stuff like that, I get it. But I mean, Maths and English doesn't really matter unless you're feeling the best anyway. So I feel like any […] mental health sort of programs or anything that can be implemented in schools is worth its weight in gold, really […] if we're going to place this emphasis on wellbeing and things like that, then [teaching] time needs to be assigned |
| Relatable, engaging and inspiring MH programs | FEMALE STUDENT_F1 | 97 | With the fires it should've been more like, You know like some of your loved ones are in the fires, it'll all be fine, it'll be alright. And with corona, they should have been like, We know you can't see everyone you love at the moment, but you have to stay away for health reasons |
|  | FEMALE STUDENT_B1 | 98 | [Referring to batyr@school program] a vulnerable person who was putting all their cards down on the table… not just like a generalised talk about mental health…’, ‘I think like because she was a young person speaking to young people, it's more relatable’ […] having that person staying they can see the vulnerability and it just, I don't know, I think it makes it more realistic and it just really, it makes me think personally, a lot more about it. |
|  | FEMALE STUDENT_B3 | 99 | [Referring to batyr@school program] And I think the fact that they talk about their lived experiences, like when someone could advertise a company, they can tell you that something is there, but when you hear someone else's story, I think that's just way more like uplifting and original when you see that stuff, how we can do it and someone else can like find some kind of escape from what they feel. That's probably the best way to reach people. |
|  | TEACHER_B4 | 100 | Someone that's actually got on the ground experience has got a far bigger impact than someone that's just gone to uni and reads something out of a book. |
|  | FEMALE STUDENT_B1 | 101 | Standardised PowerPoint and discussion, like it doesn't really work for us. We disengage, like you might just not engage and that's what we get a lot of the time. And it's the same stuff over and over again. And it doesn't register with us. |
|  | FEMALE STUDENT_F1 | 102 | getting them to involve in the discussions because everyone's sort of like mute and just listens […] it's like that lecture sort of you know, someone's up front |
|  | MALE + FEMALE STUDENT_F5 | 103 | [Referring to batyr@school program] [I: What do you think was particularly engaging about these activities?] MS1: Just little games MS2: just the way that they didn't make us sit down and like be quiet and not do anything for the whole hour MS3: a lot of the times when people come to school, I don't know they just, talk to us. But this was different. We got up and interacted with them and yeah and with each other. FS4: I feel like when they like gave us the games we listened more after that. Like we were just like more engaged after that, I guess. MS1: Wanted to be a part of it more. |
|  | MALE STUDENT_F12 | 104 | [I: Why is not being too serious important?] MS: Because it's like a symptom type, I guess, so bringing like a lightness to it makes it feel easier to understand or less confronting. |
|  | MALE STUDENT_F12 | 105 | Smaller would probably be better, 'cause it's, not everyone sort of gets contributing. It's just the louder ones. You sort of have smaller groups and everyone sort of can get involved. |
|  | FEMALE STUDENT_F3 | 106 | In our group, in our session that we had, it was Year 9 and 10 together and I think what [name of student] might be saying is, like, if we had the different like age groups or year groups in separate sessions, you could change how you present, like the talk, the change, the age group […] Change the way we deal with them, because, dare I say, a 13 year old would be a lot different to a 15 or 16 year old, I'd say. |
|  | MALE STUDENT_F3 | 107 | I think the point is […] because it was only us year 10 boys so yeah […] We just felt like we were able to be more open because it was more of a separate small group. |
|  | WELLBEING OFFICER_F8 | 108 | [Referring to batyr@school program] Due to the increasing numbers of students with mental health issues, I think I've found it to be too surface level. So it was more of just general […] if you were a person that had been suffering from significant mental health illness in that room, I don't know whether you walked away with much more than when you walked in […] Then if you go into too much detail for the other for the kids that aren't at that level, then that might have been a traumatic thing too. […] So I just wondered whether it could be tailored a little bit more […] for those kids that are at the level of significant depression, anxiety and maybe suicidal ideation level, whether they could have more of a deeper conversation […] I think it's just […] whether if moving forward, if schools identify that there is significant mental health issues within that cohort with more focus groups or mini groups would work, so they could have open conversations rather than just like sit them all together and have a delivery of that like of a, you know, of slides. |
| Empowering YP | FEMALE STUDENT_F2 | 109 | Your friends might not want to go see someone that's the hardest bit because then you don't know how to help them, but you try and do what you can. |
|  | PARENT_F9 | 110 | Siblings […] often say different things to each other than what they say to their parents […] Things that they might not be able to or want to tell their parents […] it's often peers coaching peers and talking through it, whereas it’s a different conversation where adults often lecture [inaud]. Kids listen to each other […] my daughter, who like doesn't display any mental health issues but supports a friend that does, and […] her friend won't tell her parents, but she offloads on to my daughter. |
|  | FEMALE + MALE STUDENT_F4 | 111 | [I: Can you tell me a little bit more about what you mean by being scared to help someone out?] FS: Well because, like, you might not know what to say. You might get scared of saying something or, like, you might offend them in some type of way, like, maybe and that also might scare you and especially if it's like a friend and if you say something wrong, like you might like feel like you're going to lose that friend or something. […] MS: Yeah, it’s a touchy subject. |
|  | FEMALE STUDENT_F1 | 112 | FS1: What they always do stuff like when they talk about their mental health, they always go 'it's okay to get help' but then when people reach out for help from friends and stuff, it's never like, what do you say back. […] FS2: we need more stuff on what to do. FS3: and how to help. |
|  | MALE + FEMALE STUDENT_F2 | 113 | [Referring to school-based MH programs] MS: They didn't really mention well are they our friends because as friends, we want to help our own friends. They didn't even give us any of the ways to do that. They just tell us to text to someone who is a professional. But that's not going to always help. […] FS: I feel like they needed to let us know how we could talk to a friend about it, like say if our friend had been showing symptoms. How do we actually like talk to them about it and feel comfortable around people that are older than them, because they're just feel a little bit out of place. |
|  | MALE STUDENT_B3 | 114 | I was always one to bottle up my emotions and I realized there after a while, the bottling up wasn't the best thing to do and just seeing those boys that don't normally talk today, just, then, once they talk, others started to talk and start to get what has been happening in their life out off their chest […] I'm pretty good at helping [other YP] out with those types of things because of what I've been through in my life and all that stuff. I've been through headspace, I know the sort of things on how to talk to people […] I think having students, even if it was just us five appointed for other students to talk to you, because sometimes you might not want to go to a teacher […] I realised that the teachers don't normally help as much because students don't want to talk to teachers as much. Whereas if you have different students and being the school captain I really like going up and talking to people and they more listen to their peers, and learn off their peers than most other people. |
|  | FEMALE STUDENT_F2 | 115 | We all want like someone our age to speak up about, like I tell my mom, I want to be a Batyr worker. I want to be like them. I want to be a youth mentor, something just to help other people because I've gone through it. I know what it's like. I can help people, I've helped my friends. I've helped people, I've always stuck by their sides. I always call them to make sure they're OK |
| Confidentiality and trust | MALE STUDENT_B3 | 116 | the fact that it's all online too, not a lot of people are confident with sharing things online because of the whole modern idea of hacking and all that, the security risk associated with putting stuff out on the Internet. […] the people in the area may not be talking about their problems and stuff as much because as [student] said, that the things going around these days, if you go on one of the sites on the Internet, then there's a chance it could get hacked and all your stuff could then be out there. So if you had that one-on-one personal contact with somebody and everybody knew that it was completely confidential and possibly boost people going to those type of places around this area. |
|  | TEACHER_B4 | 117 | It really takes for the kids to trust us as teachers, for them to finally come out and say something. |
| Early and ongoing preventative MH education | TEACHER_F10 | 118 | I think a more long-term program is something that may be useful. So even if it's like once a fortnight, someone comes out because then they can have set activities and tasks or things that they need to work on for their own I call it their toolkit, their mental health toolkit for those two weeks. And then when they've ticked that off and they've learned to control whatever or use this strategy, then we can add more and more. And whether it's something that's done in terms one and three or two and four […] a more long-term stable program that continues each year is definitely something that we need. |
|  | TEACHER_F11 | 119 | [Referring to batyr@school] I think we're still got a long way to go, but it's definitely, as I said, it's been the start of hopefully something pretty big and I think, I mean, I know the plan is to continue with it. So sort of build the foundation of our wellbeing processes and just positive mental health as well. So just more like a lot of prevention as well, sort of start that conversation early and get that presence in the school and the school community |
|  | PARENT_F9 | 120 | [Batyr] only sort of worked with year 9 and I thought, Oh I wish they had really worked with all the kids […] all [my children] sort of missed out. And, I thought, Damn that would have been good because I think it's good for all kids, not necessarily just one cohort and especially kids if you're in year 7, you may be struggling with fitting into a new school, or whereas in year 10, you're having depression and study or whatever's put on top of them, like work wise, time wise. I think it's good for all kids to expose to it. […] [Are you saying that you think that then the need is there for something in the primary school years?] One hundred percent. Definitely. |
|  | FEMALE + MALE STUDENT_F7 | 121 | FS1: I think [batyr@school] should have been in more years MS: It should have been all years that were spoken to […] FS: Yeah. It still needs to be said to younger kids, obviously I think [I: When would you have wanted to start hearing this kind of message?] FS: Probably as soon as I got into high school. MS: Probably as soon as I started year five […] FS: For me it would have been like high school because it's different to everything else, I guess. [I: And what about you, [student name]?] MS2: Yeah, probably year seven. It's because I had a counsellor in year five. |
|  | FEMALE STUDENT_B3 | 122 | I think we need more resources into primary, because mental illness doesn't have an age limit and a lot of younger kids aren't going to know what to do |
|  | FEMALE STUDENT_B1 | 123 | It kind of doesn't process that it's not, that's not how it has to be, like you can get help. But I don't think we realize that we need it. Like, that's what the Truth Project does. Like she was saying, that she didn't realize she needed the help then, but like looking back, she knows that she should have went and got that help earlier. |
|  | FEMALE STUDENT_B1 | 124 | I feel like most of us can't differentiate anger, from frustration or sadness from just like being a little bit upset and like even with happiness versus content. I don't think, I feel like I feel like that's something that we fail to recognize because we don't know, I guess |
|  | FEMALE + MALE STUDENT_F2 | 125 | They should have done like at least a slideshow or something about definitions of the different mental health, like say someone didn't know what bipolar was or something like that MS: Or what self-harm can do to someone […] FS: Then someone could get an actual recognition of what the actual what it actually meant and more understanding of, um, also what it meant so then, if anyone doesn't know what it is, then they'd have a definition of what it is and probably how it can be resolved and who you could go to about it. FS2: So, say someone who had bipolar, but they didn't know what it was, so and they didn't know that they had it. And then they told them the definition and the symptoms and what not and they'll be like, Oh shit I have bipolar! |
|  | MALE STUDENT_B3 | 126 | men are often expected to be doing a lot of the harder work and not talk and go on drinking on the weekends |
|  | TEACHER_F11 | 127 | obviously small rural school, if I'm going to be a bit stereotypical, like a lot of these kids come from properties or things like that where, you know, like they've, we've had a few issues with like sort of boys education here. It's just sort of that stereotypical, just want to go on the farm, that masculinity, don't want to talk about it, all that sort of stuff. So I really feel like it's yeah, it's starting to break down those walls quite a bit […] 32:35: whether it be like some sort of journaling on like, how's your week been? How are you feeling? That reflection or something like that. |

^: Participants are referred to using a unique identifier denoting gender (F/M] (for students only), participant group [S = Student, T = Teacher, WBO = Wellbeing Officer, P = Parent,] and interview session code (e.g., B3, F1). The researcher interviewing participants is referred to as I.
