# Supplementary material for: A qualitative exploration of young people’s mental health needs in rural and regional Australia: engagement, empowerment and integration
Source: BMC Psychiatry. 2023 Oct 13;23:745. doi: 10.1186/s12888-023-05209-6 (PMC10571294; doi:10.1186/s12888-023-05209-6)
Supplement: Supplementary file 5 — Additional file 5. [file 12888_2023_5209_MOESM5_ESM.docx]

**Table of Principles and key facilitators**

| **Principles** | **Key facilitators** |
| --- | --- |
| Engagement | - Relatability to engender emotional engagement - Lived experience mental health stories from other young people - In-person delivery to facilitate trust building, confidentiality and interactivity; online delivery if required to maximise reach - Local knowledge and according program tailoring |
| Empowerment | - Promoting active participation - Offering choice and self-determination - Practicing MH conversations - Strengthening guided informal, peer-based support (e.g., Mental Health Ambassadors) - Building a positive mental health culture - Including parents into education and support programs |
| Integration | - Coordinating school-based with non-school-based programs (e.g., community-based and external visiting services) - Early and ongoing integration of mental health education in school curricula - Teacher support, education and integration into mental health programs - Strengthening social connections between family, school and community - Co-design with key stakeholders (e.g., young people, parents, teachers) |
